# Transcranial Corticospinal Motor-Evoked Potentials in Cases of Ventral and Ventrolateral Intradural Extramedullary Cervical Spinal Cord Tumors

**DOI:** 10.3390/medicina60091488

**Published:** 2024-09-12

**Authors:** Petra Vasileva, Hristo Hristov, Assen Bussarsky, Rositsa Tanova, Vasil Karakostov, Dilyan Ferdinandov

**Affiliations:** 1 Clinic of Neurosurgery, St. Ivan Rilski University Hospital, 1431 Sofia, Bulgaria; 2 Faculty of Medicine, Medical University—Sofia, 1431 Sofia, Bulgaria; 3 Clinic of Anesthesiology and Intensive Care, St. Ivan Rilski University Hospital, 1431 Sofia, Bulgaria

**Keywords:** ventral or ventrolateral location, intradural extramedullary tumor, cervical spine, transcranial motor-evoked potentials, intraoperative neurophysiological monitoring

## Abstract

*Background and Objectives*: We studied the clinical significance of an amplitude decrement and disappearance alarm criteria in transcranial motor-evoked potential (MEP) monitoring during surgeries on extramedullary tumors at the cervical spine with reference to postoperative morbidity. *Material and Methods*: We diagnosed and surgically treated fourteen patients with intradural extramedullary ventral or ventrolateral lesions to the cervical spinal cord in the Clinic of Neurosurgery at the University Hospital St Ivan Rilski from January 2018 to July 2022. Eight cases were diagnosed with schwannoma, and the remaining six had meningiomas. The follow-up period for neurological assessment was six months. *Results*: A decrease in the intraoperative transcranial MEPs of 50% or more compared to baseline in two cases (14.3%) resulted in an immediate postoperative motor deficit. One patient demonstrated full neurological recovery within six months, while the other exhibited only partial improvement. In six cases (42.9%) with preoperative motor deficits, tumor resection and decompression of the cervical spinal cord led directly to an increment of the transcranial MEPs by more than 20%. Postoperatively and at the 6-month follow-up, these patients showed recovery from the preoperative deficits. In the remaining cases, MEPs were stable during surgery with no clinical deterioration of the motor function. *Conclusions*: The decremented MEP criteria corresponded to postoperative motor deficit, whereas the improvement of the same parameters after decompression implied future recovery of preoperative motor deficits. The combination of different MEP criteria is likely to be helpful when tailored to a specific case of ventral or ventrolateral extramedullary lesions in the cervical spine.

## 1. Introduction

Intradural extramedullary (IDEM) tumors are usually benign neoplasms that arise in the spinal canal. They represent around 80% of all primary lesions affecting the spinal cord and about 15% of central nervous system neoplasms [1,2,3]. The most common ones include meningiomas and schwannomas. They have a slow-growing nature, which subsequently causes considerable dislocation of neural structures and narrowing of the spinal canal with nervous tissue compression. Depending on the lesion’s size and location, the latter corresponds to the clinical manifestation. In rare cases, neoplasms can be ventral or ventrolateral to the spinal cord at the cervical and thoracic levels, causing neurological symptoms when they occupy >75% of the vertebral canal [4]. The tumor’s anatomical location and the surgeon’s restricted working space can be challenging regarding the degree of surgical resection with preservation of the spinal cord and nerve integrity [5].

As many authors agree, intraoperative neurophysiological monitoring is vital to detect possible injuries during surgical interventions and to predict postoperative morbidity and neurological deficits. This information allows the surgeon to opportunely change the resection approach and strategy to avoid permanent neurological functional damage [6,7]. The continuous monitoring of the transcranial corticospinal motor-evoked potentials (MEPs) assesses the functional integrity of the white matter corticospinal tract and the synapses of the grey matter [8], which have a prominent role in predicting the postoperative result [6]. The acceptable sensitivity and specificity of the transcranial MEPs are key factors that suggest their value in completing a maximal resection of intradural extramedullary tumor formations with maintained corticospinal tract integrity, minimized iatrogenic trauma, and better postoperative results [9,10,11]. The clinical utility of this concept for the operative treatment of the mentioned lesions is still in dispute among many studies in the literature [10,12,13,14].

We aimed to assess the role of transcranial MEPs during surgical excision for intradural extramedullary tumors ventral and ventrolateral to the spinal cord in the cervical region and their feasibility in lowering the risk of iatrogenic trauma.

## 2. Materials and Methods

A cohort of fourteen patients from a retrospective study on intraoperative neurophysiological monitoring in surgeries for intra- and extramedullary tumors is presented. All cases were diagnosed and surgically treated in the Clinic of Neurosurgery at the University Hospital St. Ivan Rilski from January 2018 until July 2022.

The inclusion criteria were surgical interventions with intraoperative transcranial MEP monitoring for ventral or ventrolateral intradural extramedullary tumors at the cervical spine under total intravenous anesthesia with propofol. The exclusion criteria were cervical degenerative stenosis or disc herniation with spinal cord compression, location of tumors in the cervicothoracic junction or below, and impossible motor-evoked potential gain, i.e., patients with a complete motor deficit. 

The cervical localization of the tumors required monitoring with transcranial MEPs from both the upper and lower extremities. The compound action potentials were recorded from the selected muscles using needle electrodes. The preferred ones from the upper extremities were the abductor pollicis brevis, the biceps brachii, and the triceps brachii. We usually choose the abductor halluces and tibialis anterior muscles for the lower extremities. Each patient was monitored on 8 channels with constant feedback from the neurosurgeon.

Intravenous anesthesia with propofol was maintained to avoid confounding effects on MEPs, and a short-acting neuromuscular blocker was used only for intubation. The transcranial stimulation was done according to the International 10–20 electroencephalogram system, with needle electrode pairs positioned at C3 and C4. Short trains of five to seven square-wave stimuli were delivered, with a duration of 1 ms, an interstimulus interval of 4 ms, and multi-pulse current stimulation with 200 mA. The responses were checked every 15 min; before and after laminectomy; during spinal cord retraction; before, during, and after tumor resection; and before the closure of the dura mater. We applied the alarm criteria to all recorded muscles, expressed in a ≥50% decrement from baseline. If the alarm criteria had occurred, the current surgery was halted, and irrigation was performed. The surgery proceeded again when the responses returned to baseline.

The motor function and muscle strength of the upper and lower extremities on each side were assessed at admission, immediately after surgery, at discharge, and six months after the intervention. The clinical outcome was evaluated according to the Medical Research Council (MRC) score for muscle strength from six proximal and distal muscle groups on both sides for the upper and lower extremities. A decrease in the postoperative MRC score of more than 1 point compared to the preoperative score was defined as a new postoperative motor deficit.

## 3. Results

The patients were from 39 to 78 years old (mean age of 62.5 years), with a prevalence of the female sex by 12 (85.7%) to two (14.3%) cases. Eight (57.1%) were diagnosed with schwannoma ventrolateral to the spinal cord in the cervical region, while the rest (42.9%) had meningiomas with ventral or ventrolateral localization, summarized in Table 1.

In two patients (14.3%), described as Case 1 and Case 2, the amplitude of MEPs decreased by more than 50% from baseline. In contrast, in other six cases, the MEPs threshold increased by more than 20% from baseline after tumor resection and spinal cord decompression. Throughout the surgical intervention, the MEPs remained stable across the remaining cases, with no immediate changes observed in the MRC score post-procedure. Despite the unaltered intraoperative MEPs, patients uniformly reported a subjective enhancement in muscular strength during the early postoperative phase. This perceived amelioration was substantiated objectively by a one-point improvement in the MRC score at the six-month follow-up evaluation.

Case 1 is a male, 64 years old, with no significant previous medical history. Initial neurological examination revealed upper limb paraparesis MRC 2/5, gait disturbances, right foot weakness MRC 4/5, and generalized truncal and lower limb numbness. Cervical spine MRI was significant for an intradural extramedullary lesion with the macroscopic appearance of schwannoma at C3–C7, ventrolateral to the spinal cord, shifting it to the right. During tumor resection, there was a ≥50% reduction in the MEPs for all extremities compared to their baseline values. The surgery was halted, and the operative field was flushed with warm saline. Retraction on the spinal cord was discontinued, which led to a moderate increase in MEPs. The decision to continue tumor resection was made, and no further decrease in MEPs was noted. After complete tumor resection and spinal cord release, a significant (more than 40%) increase in MEPs for the upper extremities was observed, which were near (85–90%) baseline levels. This did not correspond to a significant MEP recovery for the right lower limb. However, the amplitude of the left tibialis anterior potential improved. Postoperative neurological evaluation of the patient revealed a more pronounced deficit for the upper extremities MRC (1/5), and right lower-limb motor deficit with an MRC score of 2/5. At the six-month follow-up, the MRC score improved to 3/5 for the right foot and to 4/5 for the upper extremities.

**Figure 1:**
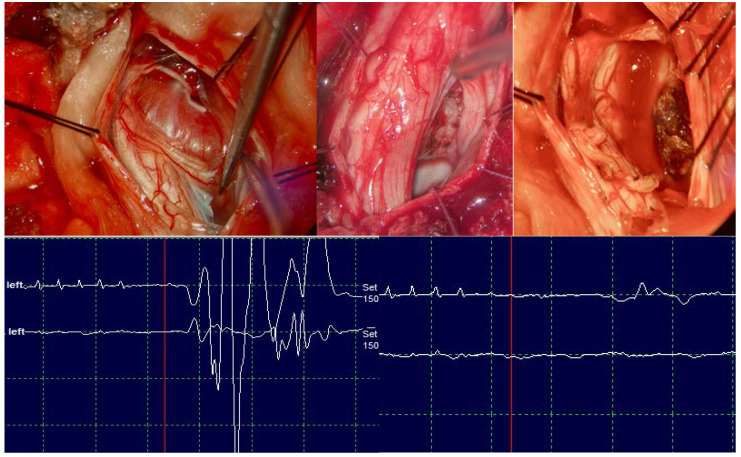
Intraoperative stages of tumor resection and MEPs reduction for the tibialis anterior muscle.

Case 2 is a female, 45 years old, and has no previous significant medical history. Initial neurological examination revealed axial pain and lower extremity and truncal numbness with no motor weakness. MRI of the cervical spine was significant for an intradural extramedullary lesion at C3–C5 with the macroscopic appearance of a meningioma (Figure 1). During surgery, significant calcifications in the tumor were encountered, and a continuous retraction of the spinal cord was necessary. Before complete removal could be achieved, a decrease in upper extremity MEPs of more than 50% and a complete disappearance of lower extremity MEPs was observed. The surgery was halted, and spinal cord retraction was released, followed by tumor debulking by an ultrasonic surgical aspirator. However, lower extremities’ MEPs did not recover at the end of the surgery, corresponding to an immediate postoperative motor deficit, with MRC scores of 4/5 for the right arm flexion and 2/5 for the lower extremities. The immediate postoperative deficits for the right arm and the lower limbs recovered entirely throughout the follow-up period of six months (MRC score of 5/5).

Five individuals (35.7%) demonstrated preoperative lower extremity paresis MRC 3/5, and one patient (7.1%) exhibited a more pronounced motor deficit with a preoperative MRC 2/5, concomitant with gait disturbances. In the early postoperative period, a one-point enhancement in MRC score was noted for all patients.

Intraoperatively, there was a notable increase of up to 20% in motor-evoked potentials following tumor excision in the six cases mentioned above (42.9%) presenting with preoperative deficits. These patients described immediate postoperative subjective improvement in the motor function of their limbs, which was corroborated objectively by an upturn in their MRC scores at discharge and upon re-evaluation after six months.

**Figure 2:**
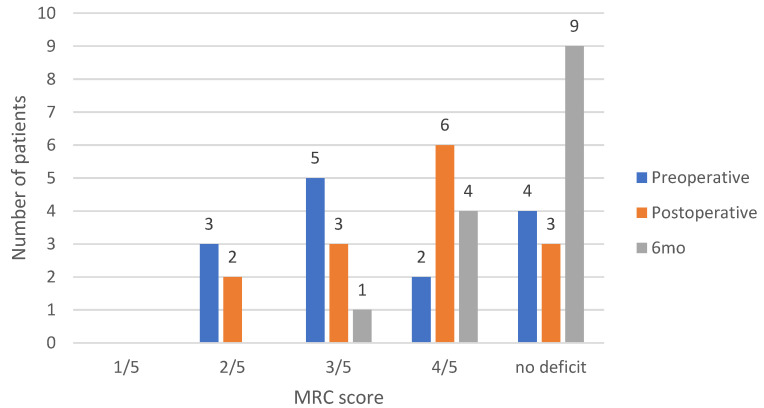
Summary of the clinical cases in the cohort with their MRC score preoperative, postoperative, and at 6-month follow-up.

During the remaining surgical interventions, the MEPs were consistently stable. In cases where patients presented with no preoperative motor deficits, the MRC score postoperatively remained unchanged, and there was no evidence of postoperative deficits upon assessment at any interval. Patients exhibiting preoperative motor weakness, with steady MEPs intraoperatively, reported subjective improvements post-surgery, which at the six-month evaluation showed an objective enhancement of the MRC score with one unit. The change in the clinical cases’ MRC score is presented in Figure 2.

For the number of patients in the presented case series, we ran the Pearson Correlation Coefficient to measure the linear correlation between the intraoperative MEPs and the MRC score in the 6-month follow-up. The variables received values in percentages, negative when decreasing and positive when increasing. After six months, the variables “intraoperative MEPs” and “clinical MRC score” were moderately positively correlated, *r* = 0.384. Thus, the result is not statistically significant since the *p*-value is 0.195. This means the study does not have enough evidence to confidently assert a true relationship between intraoperative MEPs and the MRC score in this dataset. It is to be noted that the sample size limits the statistical power, leading to less reliable estimates and making it difficult to detect statistically significant relationships, even if there is some correlation. A larger sample might provide more power to detect a significant relationship if one exists. The moderate positive correlation (*r* = 0.384) suggests that as the percentage change in MEP amplitude increases, muscle strength at the 6-month follow-up also tends to increase. However, the correlation is not strong enough to be conclusive. The statistical test suggests that the findings should be interpreted cautiously due to the small sample size.

## 4. Discussion

IONM has increasingly been integrated into spinal cord tumor surgeries to mitigate postoperative neurological deficits potentially. The concurrent utilization of epidurally recorded D-waves and motor-evoked potentials was demonstrated to be a valuable predictor of postoperative motor outcome in patients with intramedullary spinal cord tumors (ISCTs) [15,16,17]. In this regard, several studies on ISCT surgery have demonstrated that the loss of muscle MEPs in the presence of a D-wave which was observed to be up to 50% of its baseline amplitude resulted in only transient motor deficits [16,18]. The utilization of IONM with MEPs and D-waves during surgical procedures for ISCTs has become a standard practice [16,18,19,20]. As posited by Macdonald et al. [18], while invasive D-wave monitoring may be omitted in surgical procedures other than those of intramedullary tumors, transcranial MEPs indicate the integrity of the corticospinal tract and demonstrate a high degree of sensitivity to ischemic events. Furthermore, non-invasive recordings are not affected by changes in the position of the spinal cord.

The ‘all or nothing’ criteria for MEPs have been adopted as an alarming sign of intraoperative neuromonitoring of spinal cord integrity for cases of intramedullary tumors and scoliosis surgeries [18,21,22]. The primary concern regarding the ‘presence or absence’ rule is its limited informativeness and lack of sensitivity to partial damage to the corticospinal tract, which may result in postoperative neurological deficits. Such partial damages frequently manifest as a reduction in transcranial MEPs from the baseline rather than their complete disappearance [23,24]. Many authors advocate the replacement of the ‘all or nothing’ criteria with the reduction in the wave amplitude, although the precise range, from 50% to 80%, remains a topic of contention [18,21,22,25,26,27]. In a previous study, Kim et al. [28] compared the two alarm criteria mentioned above for transcranial MEPs in patients with intramedullary and extramedullary lesions in the cervical spine. The findings indicated that the 70% decrement is more sensitive for immediate motor deficit. For the six-month follow-up of the patient’s deficit, the 70% decrement and the complete intraoperative loss of MEPs demonstrated comparable sensitivity. In the context of intramedullary spinal cord tumor surgery, it has been demonstrated that non-invasive techniques alone are insufficient, and that invasive D-wave monitoring is essential for achieving optimal surgical outcomes. Nevertheless, our findings indicate that non-invasive monitoring provides sufficient reliability for postoperative deficit predictions based on amplitude decrement criteria. It should be noted that this does not preclude the use of invasive techniques as part of the monitoring protocol in tailored cases.

In 2017, a meta-analysis by Hadley et al. [29] reported only a limited number of evidence class II and III studies focusing on IONM in IDEM, with divergent statements on feasibility in preventing postoperative neurological deterioration. In their retrospective study, Harel et al. [30] compared 40 monitored IDEM tumors with 70 historical controls of IDEM lesions without the use of IONM. The authors supported that IONM is an adjunct for predicting postoperative deficit, with sensitivity, specificity, and positive and negative predicted values of 75%, 100%, 100%, and 97%, respectively. Despite this, the authors refute the therapeutic benefit of IONM in IDEM tumors. A retrospective analysis of 100 patients who underwent IDEM resection revealed that Korn et al. [10] achieved a sensitivity of 82%, a specificity of 95%, a positive predictive value of 82%, and a negative predictive value of 95% through IONM. The authors reported that significant transcranial MEP amplitude attenuation (>75%) was brought to the surgical team’s attention, leading to interventions such as pausing the procedure or altering the surgical focus. Of the 29.8% of patients who experienced significant MEP events, 48.3% saw these issues resolved intraoperatively, and 12 out of 14 patients with resolved MEP changes had no new postoperative deficits. This underscores the potential of IONM to avert long-term neurological harm promptly. Velayutham et al. [21] argued that early intraoperative correction of surgical manipulations for decreased MEP could prevent severe postoperative deterioration in limb motor function. The authors observed postoperative neurological deficits in muscles with intraoperative MEP impairment. Tumor location (intramedullary or extramedullary) did not affect the predictive value of the MEP monitoring, which had high sensitivity (100%) and specificity (98.1%) in patients with both intramedullary and extramedullary tumors.

Ghadirpour et al. [17] presented a case series of 68 patients undergoing IDEM resection over a five-year period, in which they employed IONM. In 63 cases, total resection was achieved without any monitoring events. In three cases, alterations to the monitoring plan resulted in a significant change to the surgical approach. However, the surgeons ultimately achieved complete resection in all three cases. In two cases, subtotal resection was achieved due to the occurrence of permanent potential changes, which predicted mild transient neurological deterioration. The authors posit that these five cases represent instances where IONM prevented permanent neurological damage; however, there is currently no evidence to substantiate this assertion. The results presented in the study of Harel et al. [30], along with those reported in other studies on IONM for IDEM tumor resection, indicate a monitoring event rate of 14–24%, with approximately 50% of these events resulting in new neurological deficits.

In the majority of studies examining intradural extramedullary tumors, the cervical region accounts for a relatively small proportion of cases. Mirza et al. [25] observed that the greater part of IDEM tumors was located in the thoracic region, likewise for the predominant lesion location among the total number of 68 cases described by Ghadirpour et al. [17]. In their study, cervical IDEM tumors constituted 16.18% (11 cases). Randhawa et al. [22] reviewed 130 patients, where axial and sagittal distribution in relation to the spinal cord was, respectively, 19.2% ventral and 13% cervical. Furthermore, the authors revealed an association between the location and the postoperative outcome. Specifically, thoracic tumors and ventral tumors have been found to have poorer functional outcomes than other locations. This finding was consistent with the results of other studies [23], which have also demonstrated that tumors in the thoracic and ventral regions tend to have poorer postoperative outcomes. Accordingly, the potential correlation between changes in transcranial MEPs and tumors situated ventral or ventrolaterally to the spinal cord necessitates further investigation.

The predominant ventral and ventrolateral intradural spinal tumors are represented by schwannomas and meningiomas, which corresponds to the histological findings of other authors [10,17,22,24]. In their investigation into the contingency of MEPs’ reversible or irreversible changes in surgeries for the two most typical IDEM tumors, Mirza et al. [25] stated that both anterior schwannoma and meningioma patients express a higher likelihood of irreversible changes and, consequently, a higher risk of postoperative deficit.

The optimal treatment for IDEM tumors is their gross-total excision [22,24,26,27], with many authors preferring the dorsomedial approach. Anterolateral approaches for such tumors pose a significant technical problem due to intense bleeding from the epidural venous plexus, which impairs visual control during surgery. Difficulties also arise in planning adequate bone resections for the complete visualization of these tumors when performing anterior approaches, as well as the subsequent need for vertebrodesis.

All patients from the current case series presented with anterior and anterolateral lesions dislocating the cervical spinal cord to the contralateral side underwent tumor resection through a posterior approach. A laminectomy after verification of the level of interest was performed, with the articular facets undermined for better visualization and minimal retraction of the nervous tissue. After the midline incision of the dural sheath, to promote mobility and to minimize iatrogenic trauma to the spinal cord, the denticulate ligaments superior and inferior to the tumor level are transected. In this way, the field of vision is expanded without significant spinal cord retraction. This maneuver is supported by other authors [24,26], who achieved complete anterior tumor resection and proved the feasibility of the dorsal approach over the extreme lateral or anterior approaches advocated by Slin’ko and Al-Qashqish [27].

Despite the gentle mobilization and minimal retraction of the spinal cord obtained with the described surgical techniques, there is still a risk of spinal cord damage in ventral meningiomas with calcifications, with no clear demarcation from the spinal cord tissue. This was observed in the two patients with such tumor specifics from our series. The MEP attenuation led to expected clinical deterioration in the immediate and short-term postoperative period. However, in the follow-up period, one of the patients demonstrated a complete MRC score recovery and a corresponding lack of permanent motor deficit. Conversely, the other patient showed only partial recovery with one unit of the MRC score (4/5). Both cases may be a result of corticospinal tract contusion during tumor adhesiolysis and spinal cord alleviation from compression, which in one patient demonstrated motor function regain after some time needed for fiber recovery. Similar results are reported by Harel et al. [30] for postoperative deficits in three patients with anterior and anterolateral tumors, and the authors explained the IONM changes with expected spinal cord excessive manipulation in ventral tumors with adhesions to the spinal cord. They argue that despite MEPs’ deterioration, gross total surgical resection should be accomplished, since the patient would not benefit from IDEM tumor subtotal excision. Korn et al. [10] emphasize that IONM MEPs attenuated in eight of the 13 patients with significant tumor–cord adhesions, concluding the correlation between the two. This may be attributed to the anterior spinal vasculature and the anterolateral corticospinal tract. Transcranial MEPs can give constant feedback to the neurosurgeon on the status of the corticospinal tract function. Therefore, intraoperative alarm criteria for an MEP decrement or disappearance have a role in predicting immediate or future motor deficits.

This study aimed to represent the feasibility of the transcranial MEP alarm criteria in cases of ventral or ventrolateral cervical spine tumors for postoperative motor deficits. We have marked the tendency of improving muscle function after decompression, which was confirmed intraoperatively by the increment of the MEPs. The postoperative deficit observed in cases of partial or complete MEP disappearance indicates the potential for enhanced IONM utility in this specific subgroup.

It is important to recognize two principal limitations within the scope of this investigation. The first limitation stems from the fact that the sample size was relatively small. This constraint in subject number may be attributable to the specific tumor properties under study—namely, ventral or ventrolateral localization and spinal cord adhesions and/or unclear tissue demarcation. The second limitation pertains to the exclusion of the D-wave from the spinal cord monitoring protocol. Despite the D-wave being one of the premier metrics for spinal cord monitoring, its application was deemed unnecessary due to the extramedullary location of the tumors and was further impeded by technical constraints. In addition, the study did not undertake a comparative analysis involving different spinal pathologies, for instance, juxtaposing cases with intramedullary cervical spine tumors, which could have provided broader insights.

## 5. Conclusions

According to our study results, we can conclude that there is a tendency for an improvement in the MEPs’ amplitude after the complete resection of lesions and decompression of the cervical spinal cord, resulting in an immediate postoperative and stable recovery of motor function in the 6-month follow-up. The immediate postoperative motor deficits, preceded by more than 50% decrement in MEPs rather than complete intraoperative disappearance, likely result from partial spinal cord injury due to retraction or manipulation, particularly in cases involving adhesions of anterior or anterolateral tumors to the spinal cord. These deficits typically exhibit recovery within 3–6 months. Correlations between IONM changes and clinical outcomes support the assumption that surgical feedback indicating electrical changes in MEPs may contribute to preserving the patient’s neurological status. IONM in surgeries for IDEM tumors is feasible and valid and may assist in identifying possible adverse deficits. Conversely, the efficacy of IOM in the context of IDEMs remains to be substantiated since it fails to reduce the risk of neurological deterioration and thus has not become an established standard of care.

## Figures and Tables

**Table 1:** Summary of the clinical cases in the cohort.

Gender, Age	Region	Diagnosis	Clinical Presentation	TcMEPs Changes	MRC Score Postoperatively	MRC Score in 6 Months
Female, 78	C4–C5	Schwannoma	LE paraparesis (3/5); numbness in UE	A 20% improvement from baseline of LE MEPs; No change from baseline of UE MEPs	Improvement of LE paraparesis to 4/5	Improvement of LE paraparesis to 5/5
Female, 70	C1–C2	Schwannoma	No motor deficit; cervical pain	No change from the baseline	Unchanged	Unchanged
Female, 68	C1-C2	Schwannoma	No motor deficit; R UE numbness	No change from the baseline	Unchanged	Unchanged
Female, 76	C6–C7	Meningioma	Paresis in R TB 1/5; LE paraparesis 2/5	A 20% improvement for LE MEPs from the baseline; No change from R TB MEPs	Improvement of LE paraparesis to 4/5; Improvement of R TB paresis to 4/5	Improvement of LE paraparesis to 5/5; and R TB to 5/5
Female, 70	C7	Schwannoma	Cervical pain; LE paraparesis 2/5	A 20% improvement for LE MEPs from baseline; No change of UE MEPs	Improvement of LE paraparesis (3/5)	Improvement of LE paraparesis to 4/5
Male, 64	C3–C7	Schwannoma	UE paraparesis 2/5; R LE paresis 4/5	A 50% drop of MEPs for UE and LE; MEPs for UE recover to baseline; No complete recovery to baseline for R TA MEPs	UE paraparesis 1/5; R LE paresis 2/5	Improvement of UE paraparesis to 4/5; R LE paresis 3/5
Female, 45	C3–C5	Meningioma	Cervical pain, Numbness in LE; No motor deficit	A 50% drop of MEPs for UE and complete loss for LE; MEPs for UE recovered nearly to baseline; No recovery to baseline for LE MEPs	R BB paresis 4/5; LE paraparesis 2/5	Recovery of UE and LE MRC score to 5/5
Female, 69	C7	Schwannoma	Paraparesis for UE 3/5; R LE 1/5 and L LE 2/5	A 20% improvement from baseline	Improvement of UE paraparesis 4/5 and LE paraparesis 3/5	Improvement of UE and LE MRC to 4/5
Female, 52	C4-C7	Meningioma	Cervical pain; No motor deficit	No change from the baseline	Unchanged	Unchanged
Female, 60	C7	Meningioma	L UE paresis 3/5; R UE paresis 4/5; L LE paresis 3/5	A 20% improvement for the MEPs L UE and LE; Stable MEPs for R UE and LE	Improvement for L side hemiparesis 4/5 and of R UE 5/5	Improvement of L side hemiparesis 5/5
Male, 39	C4-C5	Schwannoma	L UE paresis 4/5; R BB paresis 3/5; LE paraparesis 4/5	No change from the baseline	Subjective improvement of muscle strength	Improvement of UE and LE MRC to 5/5
Female, 55	C6-C7	Meningioma	R UE paresis 4/5	No change from the baseline	Subjective improvement of muscle strength	Improvement of R UE MRC to 5/5
Female, 70	C6-C7	Schwannoma	UE paraparesis 4/5; LE paraparesis 3/5	No change from the baseline	Subjective improvement of muscle strength	Improvement of UE paraparesis to 5/5 and of LE to 4/5
Female, 60	C7	Meningioma	Cervical pain and C7 radiculopathy; LE paraparesis 3/5	A 20% improvement for MEPs for LE; No change from the baseline for UE MEPs	Improvement for LE MRC to 4/5	Improvement for LE MRC to 4/5

Abbreviations: AH = abductor hallucis muscle; APB = abductor pollicis brevis muscle; BB = biceps brachii muscle; TB = triceps brachii muscle; TA = tibialis anterior muscle; MEP = motor-evoked potential; MRC = Medical Research Council; R = right; L = left; LE = lower extremity; UE = upper extremity. MEP recordings were performed on all of the muscles mentioned above.

## Data Availability

All datasets generated and analyzed during the current study are presented in this article. Further information regarding the subjects is available from the corresponding author on reasonable request due to the patients’ confidentiality and privacy concerns.

## Abbreviations

IDEM = intradural extramedullary; ISCTs = intramedullary spinal cord tumors; MEPs = motor-evoked potentials; MRC = Medical Research Council.
